# Room-Temperature CO_2_ Hydrogenation to Methanol over Air-Stable hcp-PdMo Intermetallic Catalyst

**DOI:** 10.1021/jacs.2c13801

**Published:** 2023-03-30

**Authors:** Hironobu Sugiyama, Masayoshi Miyazaki, Masato Sasase, Masaaki Kitano, Hideo Hosono

**Affiliations:** † MDX Research Center for Element Strategy, International Research Frontiers Initiative, Tokyo Institute of Technology, Yokohama 226-8503, Japan; ‡ Advanced Institute for Materials Research (WPI-AIMR), Tohoku University, Sendai 980-8577, Japan; § International Center for Materials Nanoarchitectonics (WPI-MANA), National Institute for Materials Science (NIMS), Tsukuba, Ibaraki 305-0044, Japan

## Abstract

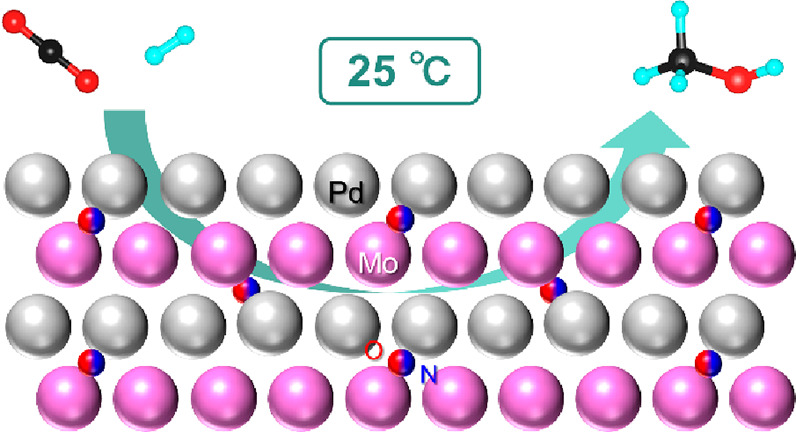

CO_2_ hydrogenation to methanol is one of the most promising routes to CO_2_ utilization. However, difficulty in CO_2_ activation at low temperature, catalyst stability, catalyst preparation, and product separation are obstacles to the realization of a practical hydrogenation process under mild conditions. Here, we report a PdMo intermetallic catalyst for low-temperature CO_2_ hydrogenation. This catalyst can be synthesized by the facile ammonolysis of an oxide precursor and exhibits excellent stability in air and the reaction atmosphere and significantly enhances the catalytic activity for CO_2_ hydrogenation to methanol and CO compared with a Pd catalyst. A turnover frequency of 0.15 h^–1^ was achieved for methanol synthesis at 0.9 MPa and 25 °C, which is comparable to or higher than that of the state-of-the-art heterogeneous catalysts under higher-pressure conditions (4–5 MPa).

Climate change and the depletion of fossil fuels are two major problems we have faced in recent years, and thus a reduction of greenhouse gas emissions into the atmosphere and the search for alternative carbon sources for energy and chemicals are urgent issues.^1^ The development of conversion processes from CO_2_, a representative greenhouse gas, to valuable chemicals is a solution to both problems, and thus the exploration of catalysts for CO_2_ utilization is accelerating worldwide.^2−4^ Methanol is one of the most promising conversion targets for CO_2_ because it can be used as a raw material for various fine chemicals, as a fuel additive, and as an energy carrier.^5,6^ Due to similarities with the industrial syngas-to-methanol process, Cu-based and Pd-based catalysts have been investigated under high-temperature and -pressure conditions (generally 200–300 °C and 3–10 MPa) for CO_2_ hydrogenation to methanol.^7^ CO_2_ hydrogenation to methanol is an exothermic reaction (CO_2_ + 3H_2_ → CH_3_OH + H_2_O, Δ*H*_298 K_ = −49.4 kJ mol^–1^); therefore, a high reaction temperature is thermodynamically unfavorable. The development of catalysts that enable low-temperature operation has thus been vigorously pursued.

Although several success stories of low-temperature methanol synthesis (≤100 °C) over homogeneous catalysts have been reported,^8−10^ heterogeneous catalysts are preferable for practical applications over homogeneous catalysts, with respect to product separation, catalyst stability, and the complexity of catalyst preparation.^5,11^ These requirements have led to active research on heterogeneous catalysts for low-temperature methanol synthesis in recent years.^12−17^ Consequently, heterogeneous catalysts that are active even at room temperature (≤30 °C) have recently begun to be reported.^18,19^ There is no doubt that CO_2_ conversion at room temperature is attractive because it requires no heat source at all; however, the conversion efficiency of these catalysts is extremely low, and therefore much more active catalysts are required for practical use.

Here we report that a PdMo intermetallic, which was prepared via a facile ammonolysis process, works as an efficient and stable catalyst for low-temperature CO_2_ hydrogenation. The catalytic performance of the PdMo catalyst was significantly improved compared with a Pd catalyst, which resulted in the realization of continuous methanol synthesis at room temperature.

PdMo catalysts were prepared via ammonolysis of the oxide precursor. Figure 1a shows X-ray diffraction (XRD) profiles of the PdMo catalysts with different Pd/Mo ratios. At low Pd content in the precursor, Mo_2_N was formed as the main phase. As the Pd content increased, another phase with the XRD pattern close to hcp-PdMo intermetallic (Supplementary Note 1) appeared and almost a single phase was obtained at a Pd/Mo ratio of 1.08. HAADF-STEM observations of the single-phase sample (Pd/Mo = 1.08) revealed an ordered structure with alternating layers of Pd and Mo, which suggests that the obtained sample is an intermetallic compound (Figure 1b and Figure S2). The results of compositional analysis of the unknown phase using the single-phase sample are summarized in Table S1. Electron probe microanalysis (EPMA) revealed that the unknown phase consists of Pd (58.1 ± 3.1 wt %), Mo (39.2 ± 3.3 wt %), N (1.1 ± 0.4 wt %), and O (1.6 ± 0.6 wt %). For Pd and Mo, a similar composition (Pd = 51.3 wt % and Mo = 44.5 wt %) was confirmed for the solution of this phase by inductively coupled plasma atomic emission spectroscopy (ICP-AES). Energy dispersive X-ray spectroscopy (EDX) mapping of a single particle showed that Pd and Mo were homogeneously distributed (Figure 1c). The amount of anions (N ≈ 2.0 wt % and O = 1.8 wt %) was also estimated by temperature-programmed desorption (TPD) measurements (Figure 1d and Figure S3), and CHN elemental combustion analysis. From these results, the elemental composition of the unknown phase was estimated to be Pd_0.6_Mo_0.4_N_0.1_O_0.1_. Based on these results, the unknown phase was tentatively identified as h-PdMo. After TPD measurement, this phase was decomposed to Pd and Mo (Figure S4), which suggests that incorporated nitrogen contributes to the stabilization of this phase. This instability is in agreement with the metastability of h-PdMo in the Pd–Mo system. Below the nitrogen desorption temperature (ca. 400 °C), this phase is thermally and chemically stable and therefore is not decomposed, even when handled in air for a long period of time (Figure 1e). Such robustness is very important when considering the practicality of a catalyst.

**Figure 1:**
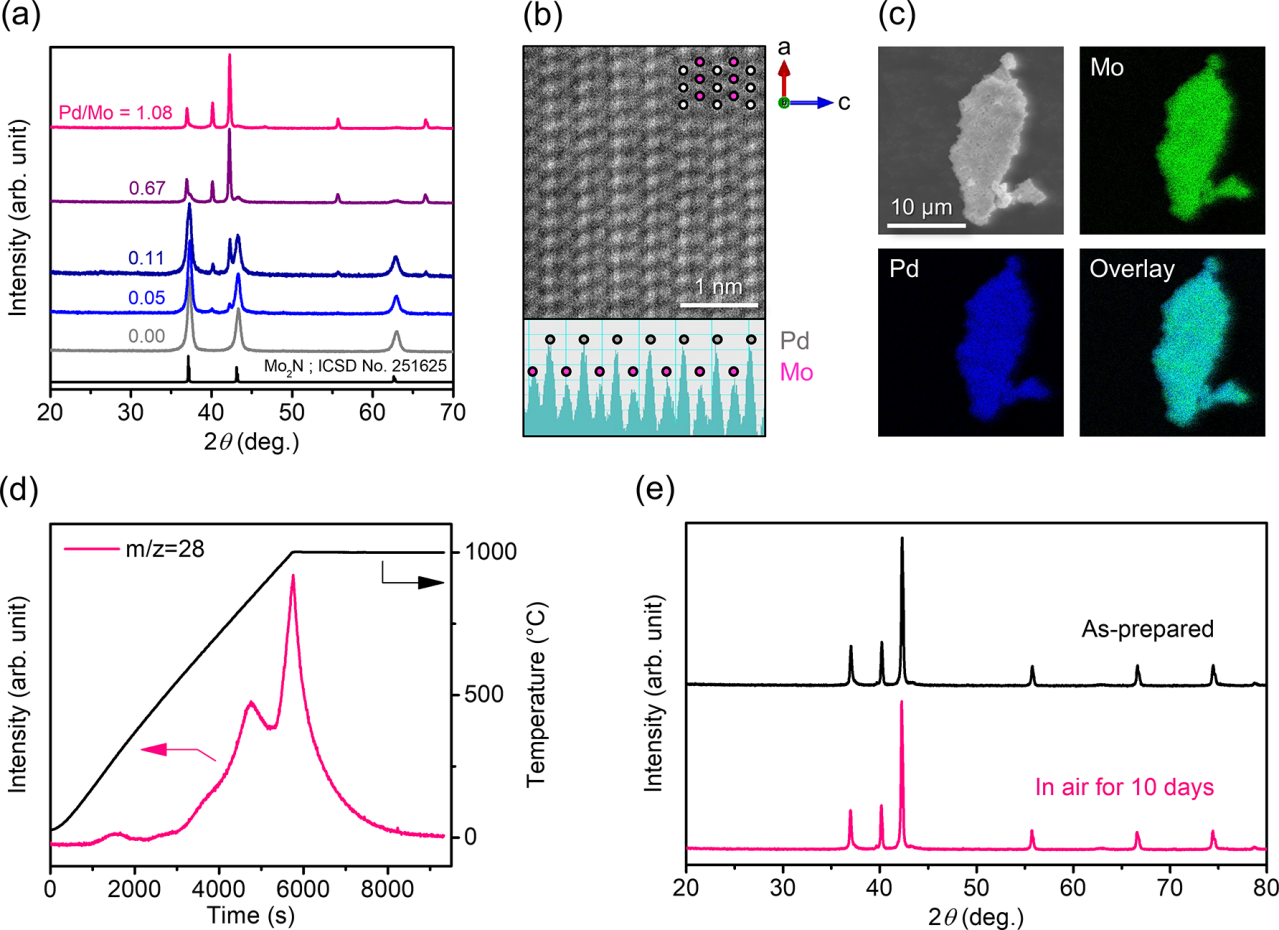
Structural characterization of PdMo catalysts. (a) XRD patterns for prepared catalysts with various Pd/Mo ratios. (b) STEM Z-contrast image obtained in HAADF mode. (c) SEM image and EDX elemental maps of Mo (green) andPd (blue) and an overlay map. (d) N_2_-TPD profile. (e) XRD patterns for h-PdMo catalyst before and after exposure to air.

h-PdMo/Mo_2_N (Pd/Mo = 0.05) was next investigated as a catalyst for CO_2_ hydrogenation in comparison with Pd nanoparticles supported on Mo_2_N (Pd/Mo_2_N) (Figure S5). According to the ICP-AES analysis, the actual amounts of Pd (4.4 and 4.6 wt %) in these two catalysts were comparable (Table S2). To confirm the state of Pd on both catalysts, microstructural observation and compositional mapping were conducted using scanning transmission electron microscopy (STEM) with EDX (Figure 2). The STEM images showed that nanoparticles were loaded on the support in both catalysts (Figure 2a,f). The distribution of Pd in h-PdMo/Mo_2_N overlapped with the regions of strong Mo and N intensity (Figure 2b–d), which indicates the formation of h-PdMo intermetallic nanoparticles attached onto Mo_2_N (Figure 2e). On the other hand, Pd in Pd/Mo_2_N was distributed separately from Mo and N (Figure 2g–i), which indicates that nanoparticles consisting of only Pd were loaded onto Mo_2_N (Figure 2j). Fast Fourier transform (FFT) pattern analyses for each nanoparticle further supported these results (Figures S6 and S7).

**Figure 2:**
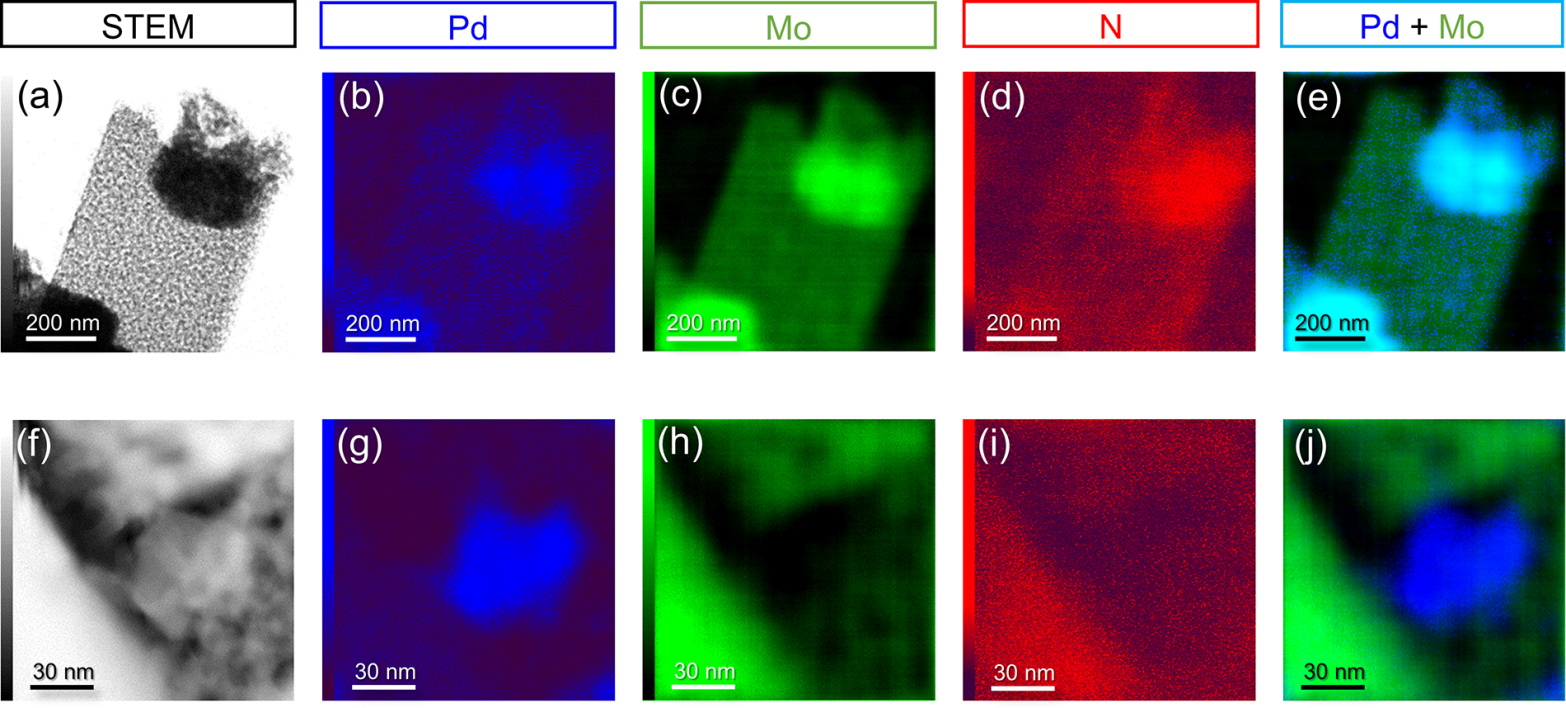
STEM observations of h-PdMo/Mo_2_N (a–e) and Pd/Mo_2_N (f–j). (a, f) STEM images and EDX elemental maps of (b, g) Pd, (c, h) Mo, and (d, i) N and (e, j) overlay maps of Pd and Mo.

Figure 3a compares the methanol synthesis activity for CO_2_ hydrogenation over the h-PdMo/Mo_2_N and Pd/Mo_2_N catalysts at ambient pressure. The catalytic activity of h-PdMo/Mo_2_N was significantly enhanced and methanol was thus produced even at low temperatures below 100 °C, whereas Pd/Mo_2_N showed some activity at elevated temperatures but no measurable activity at such low temperatures. The catalytic performance of the h-PdMo/Mo_2_N catalyst is reproducible, with a standard deviation below ±3 μmol g^–1^ h^–1^ (Figure S8). No methanol production was observed from temperature-programmed reaction of H_2_ (H_2_-TPR) on the h-PdMo/Mo_2_N catalyst (Figure S9). The h- PdMo/Mo_2_N (Pd/Mo = 0.05) catalyst synthesized by using Pd(NH_3_)_4_Cl_2_·H_2_O as the Pd source also showed catalytic activity similar to that derived from Pd(CH_3_COO)_2_ (Figure S10). These results confirmed that the methanol production was not due to hydrogenation of organic residues (e.g., Pd(CH_3_COO)_2_) but due to the catalytic reaction. Mo_2_N alone showed negligible catalytic activity, and h-PdMo showed activity similar to that of h-PdMo/Mo_2_N (Figure S11a,b); therefore, Mo_2_N itself works as a support to disperse h-PdMo nanoparticles, and the number of active sites exposed on the surface of h-PdMo is comparable to that on h-PdMo/Mo_2_N. The Cu/ZnO/Al_2_O_3_ catalyst, one of the benchmark catalysts for CO_2_ hydrogenation to methanol, also shows low activity under such low-temperature conditions, despite containing more active metal (Cu = 58.1 wt %) than h-PdMo/Mo_2_N (Pd = 4.4 wt %) (Figure S12). The apparent activation energy for h-PdMo/Mo_2_N was 27 kJ mol^–1^, which is less than half that for Pd/Mo_2_N (78 kJ mol^–1^) (Figure 3b) and the lowest among the other Pd-based catalysts reported to date (37–84 kJ mol^–1^) (Table S3). To confirm the effect of diffusion resistance on the catalytic activity, methanol synthesis activity was investigated at different flow rates when *W*/*F* (catalyst weight/flow rate) is fixed at a constant value. As shown in Figure S13, methanol synthesis rates are constant regardless of the flow rate, indicating that the reaction on h-PdMo/Mo_2_N catalyst is in kinetic control rather than diffusion control. The stability of the catalyst was also examined by a long-term continuous reaction test. Even under the low-temperature condition, the h-PdMo/Mo_2_N catalyst produced methanol continuously without degradation over 100 h at 100 °C (Figure 3c), and there was no significant change in the crystal structure before and after the reaction (Figure 3d), which indicates that h-PdMo/Mo_2_N is a stable catalyst under CO_2_ hydrogenation conditions. Similar results were obtained for the h-PdMo catalyst (Figure S11c,d).

**Figure 3:**
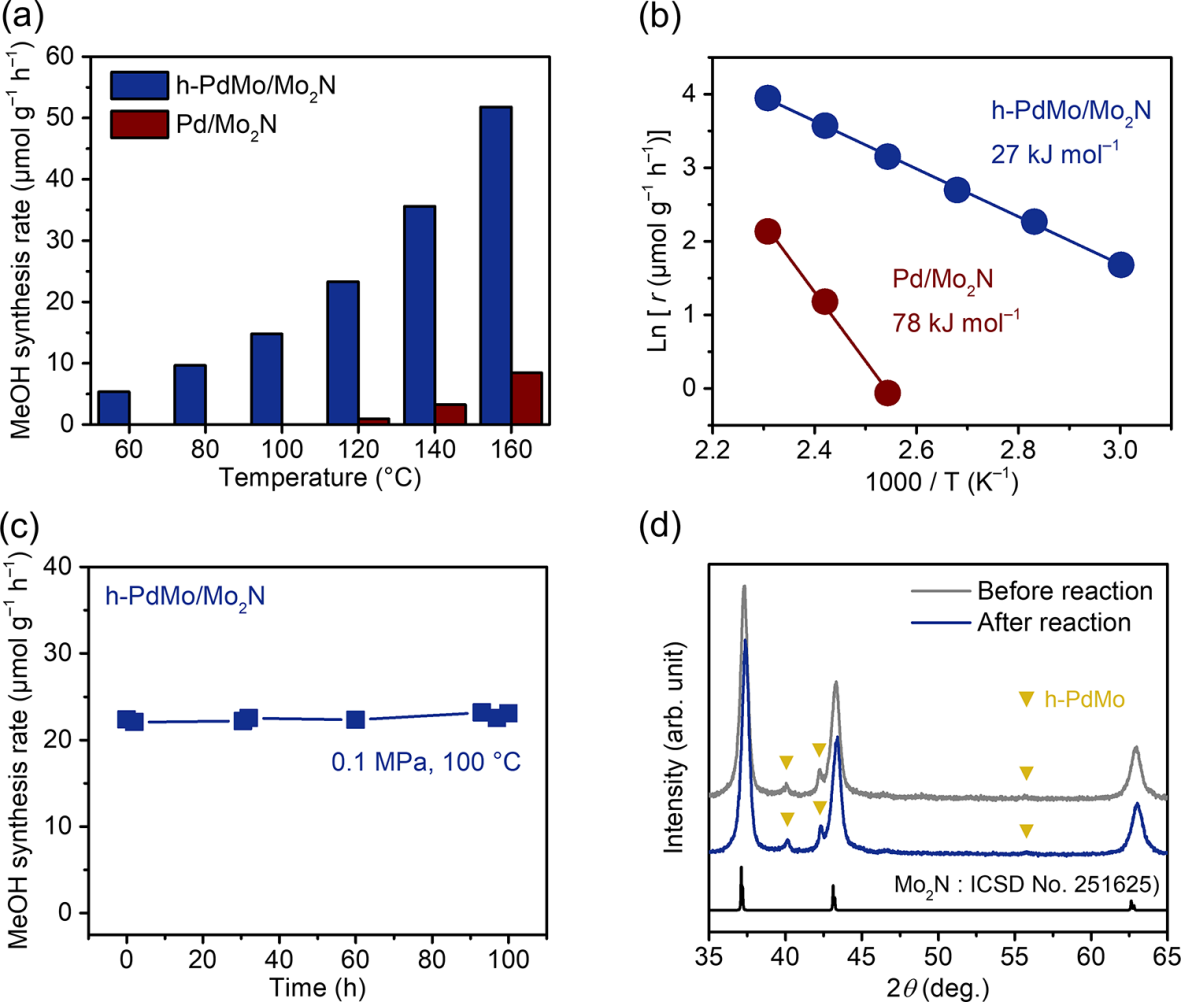
CO_2_ hydrogenation to methanol over the h-PdMo/Mo_2_N catalyst under atmospheric pressure. (a) Methanol synthesis rate as a function of reaction temperature. (b) Arrhenius plots for methanol synthesis. (c) Time course at 100 °C. (d) XRD patterns before and after methanol synthesis at 100 °C for 100 h.

From a chemical equilibrium perspective, CO_2_ hydrogenation to methanol is more favorable under higher-pressure conditions.^5^ Therefore, the catalytic performance over h-PdMo/Mo_2_N catalyst was also investigated under pressurized conditions up to 0.9 MPa with the aim of increasing the catalytic activity. Methanol synthesis activity over the h-PdMo catalyst was consequently improved with an increase of the reaction pressure (Figure S14). Figure 4a shows the temperature dependence of catalytic activity at 0.9 MPa. It should be noted that methanol was produced, even at room temperature (25 °C). The formation of isotope-labeled methanol (^13^CH_3_OH, *m*/*z* = 33) from ^13^CO_2_ and H_2_ was also confirmed under similar reaction conditions (Figure S15). h-PdMo alone (single-phase PdMo) also showed catalytic activity similar to that of h-PdMo/Mo_2_N, and their apparent activation energies were in the range of 26–28 kJ mol^–1^, regardless of the different pressure conditions (Figure 4a and Figure S16). These results suggest that h-PdMo effectively activates CO_2_ to produce methanol at low temperatures under pressurized conditions. The long-term continuous reaction test revealed that methanol was produced catalytically, even at room temperature (Figure 4b), without degradation of the h-PdMo phase (Figure S17). Assuming that all the metals on the surface of the h-PdMo catalyst are active sites, the turnover frequency (TOF) of h-PdMo was estimated to be 0.15 h^–1^ (0.9 MPa, 25 °C), although the actual number of active sites is less than that, and thus the calculated TOF value should be an underestimate. To obtain further insight into the CO_2_ activation and hydrogenation over h-PdMo/Mo_2_N at room temperature, observation of reaction intermediates in CO_2_ hydrogenation was performed by *in situ* diffuse reflectance infrared Fourier transform (DRIFT) spectroscopy measurements (Figure 4c). When the reaction gas (CO_2_:H_2_ = 1:3) was introduced, CO* and CH_3_O* species (* represents surface adsorbates) were observed as reaction intermediates. The infrared peaks at 2057 and 2076 cm^–1^ are assigned to linearly absorbed CO* species, and the peaks at 1051, 2852, and 2925 cm^–1^ are assigned to a C–O stretching vibration ν(CO) and C–H symmetric and asymmetric stretching vibrations ν(CH) of CH_3_O* species, respectively.^19−21^ The peak positions of the adsorbed species on the h-PdMo/Mo_2_N catalyst were close to those on Mo site of the reduced MoS_2_ catalyst (CO*, 2078 cm^–1^; ν(CH) of CH_3_O*, 2846 and 2915 cm^–1^)^19^ rather than those on Pd metal surface (CO*(linear): 2083–2091 cm^–1^, ν(CH) of CH_3_O*: 2860 and 2960 cm^–1^).^21−23^ In addition, CO-TPD indicates that CO is more strongly adsorbed on h-PdMo than on Pd (Figure S18). Therefore, CO* and CH_3_O* species are considered to be adsorbed on the Mo site rather than Pd site. These characteristic peaks of CH_3_O* were also observed over h-PdMo alone, and the peak intensities increased with the temperature (Figure S19), which indicates that CH_3_O* is an intermediate in CO_2_ hydrogenation to methanol over h-PdMo catalysts. The time course of intermediate formation implies that the decomposition of CO_2_ to CO* occurs first, and CO* is subsequently hydrogenated to form CH_3_O*. These results clearly demonstrate that CO_2_ activation and hydrogenation proceed over the h-PdMo catalysts, even under ambient-pressure and room-temperature conditions.

**Figure 4:**
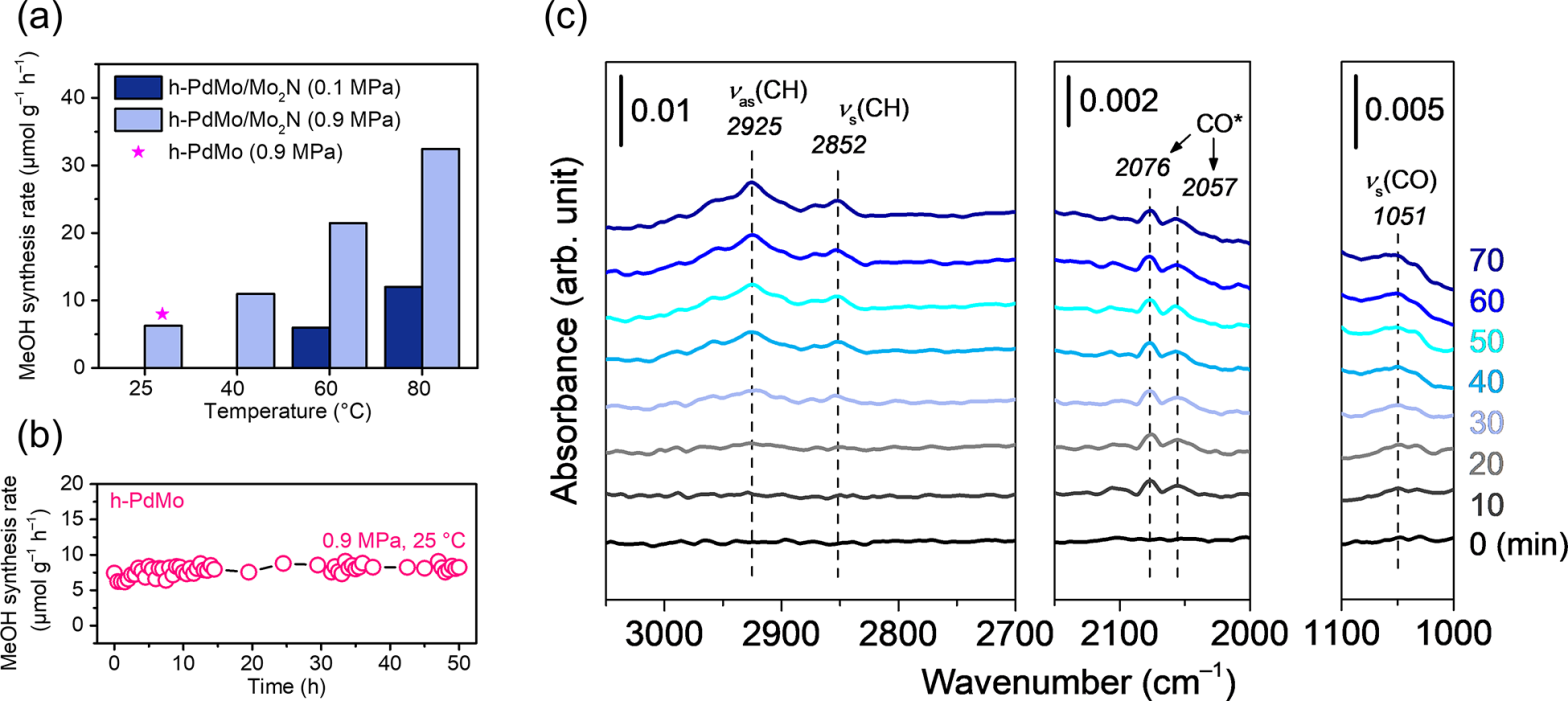
CO_2_ hydrogenation to methanol over the h-PdMo/Mo_2_N catalyst at room temperature. (a) Temperature dependence of methanol synthesis rate at 0.1 and 0.9 MPa. (b) Time course at 0.9 MPa and 25 °C for 50 h. (c) DRIFT spectra of CO_2_ hydrogenation over the h-PdMo/Mo_2_N catalyst at room temperature.

There are two major reaction pathways for CO_2_ hydrogenation to methanol: (I) *formate* pathway and (II) reverse water-gas shift (RWGS: CO_2_ + H_2_ → CO + H_2_O) and subsequent CO hydrogenation pathway (R&C pathway).^24−26^ The DRIFT spectroscopy results (Figure 4c) indicate that CO_2_ hydrogenation to methanol over h-PdMo appears to proceed via the R&C pathway. Indeed, h-PdMo/Mo_2_N catalyst also enhanced methanol synthesis from CO and H_2_, and the apparent activation energies for CO and CO_2_ hydrogenation to methanol over h-PdMo/Mo_2_N catalyst were very close, which is generally observed in Pd-based catalysts because the R&C pathway is energetically favorable (Figure 3b and Figure S20).^26^ The Pd/Mo_2_N catalyst showed a similar tendency. These results strongly suggest that CO_2_ hydrogenation to methanol on the h-PdMo/Mo_2_N catalyst proceeds via the R&C pathway. The comparable activation energies for CO and CO_2_ hydrogenation on the h-PdMo/Mo_2_N catalyst suggest that the CO hydrogenation process is rate-determining for CO_2_ hydrogenation to methanol. The rate-determining step for CO hydrogenation to methanol on the Pd catalyst is known to be the HCO formation step (CO + H → HCO).^27^ As shown in Figure 4c, the CO peak for h-PdMo/Mo_2_N catalyst is red-shifted as compared to that for conventional Pd catalysts.^21−23^ Therefore, the adsorbed CO on the h-PdMo/Mo_2_N catalyst is more activated than on Pd, which is favorable for promotion of HCO formation.^28^ The difference in activation energy between the h-PdMo/Mo_2_N and Pd/Mo_2_N catalysts can be attributed to the promotion of HCO formation on the h-PdMo/Mo_2_N catalyst.

On the h-PdMo/Mo_2_N, noticeable CO production was observed at temperatures above 100 °C (Figure S21a). CH_4_ formation was also observed at temperatures above 140 °C (Figure S21b), which suggests that hydrogenation proceeds more readily than on Pd/Mo_2_N. Selectivity of methanol over the h-PdMo/Mo_2_N catalyst decreased with increasing reaction temperature but was clearly higher than that over the Pd/Mo_2_N catalyst at temperatures below 140 °C (Figure S21c,d). Given that the hydrogenation of CO to HCO is the rate-determining step, it is difficult to suppress the CO production in the R&C pathway. However, the methanol selectivity would be much improved by enhancing the CO adsorption and low-temperature hydrogenation capability of the h-PdMo catalyst.

Finally, the catalytic activity of the h-PdMo catalyst was compared with those of previously reported room-temperature methanol synthesis catalysts.^18,19^Figure 5 summarizes the TOFs for CO_2_ hydrogenation to methanol over the h-PdMo catalyst under various reaction conditions, along with those of the reported catalysts. Comparing the catalytic performance of these catalysts at room temperature, the activity of the h-PdMo catalyst was 1 order of magnitude higher than that of the Ir complex catalyst (at 4 MPa) and comparable to that of the few-layered MoS_2_ (FL-MoS_2_) catalyst (at 5 MPa), despite the reaction pressure of <1 MPa. Furthermore, when compared under the same reaction condition (ca. 1 MPa, 60 °C), the TOF of the h-PdMo catalyst was more than 50 times higher than that of the Ir complex catalyst. In addition, the TOF value of 0.15 h^–1^ for the h-PdMo catalyst (0.9 MPa, 25 °C) is comparable to the TOF values of 0.05–0.33 h^–1^ for Cu- and Pd-based catalysts under harsh conditions (2–7 MPa, 100–250 °C) (Table S4). It should be noted that the activity of the h-PdMo catalyst increased at higher pressure, as shown in Figure S14, so that even higher activity could be expected under reaction conditions above 1 MPa.

**Figure 5:**
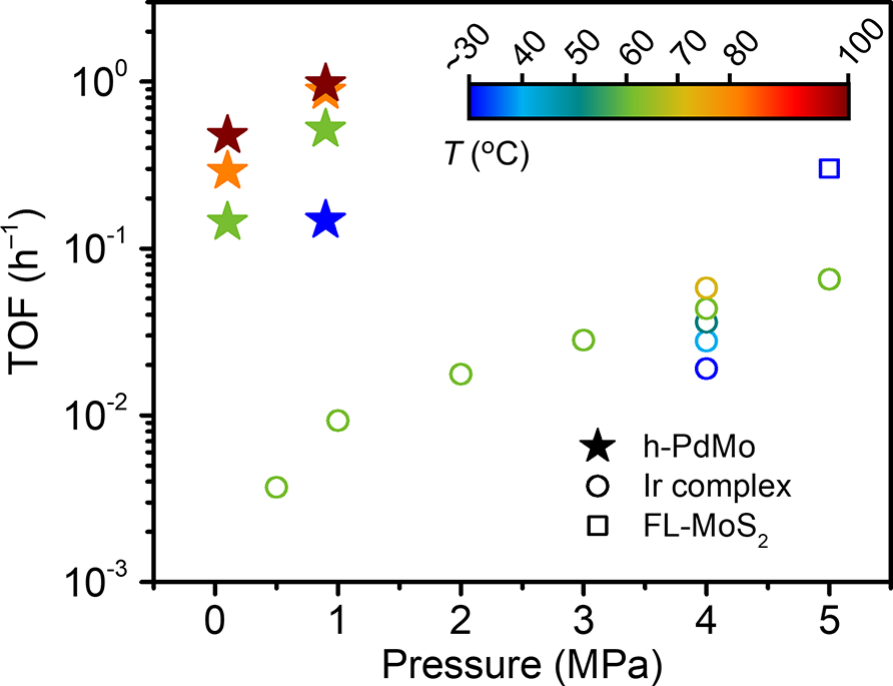
Comparison of TOFs over the h-PdMo catalyst and other reported room-temperature methanol synthesis catalysts.^18,19^

In summary, we have created a highly active and stable PdMo intermetallic catalyst, h-PdMo, and achieved room-temperature methanol synthesis from CO_2_ and H_2_. The h-PdMo catalyst can be prepared via the facile ammonolysis of an oxide precursor, and the catalyst exhibits long-term stability in air. These features are favorable for the practical use of the catalyst. For low-temperature CO_2_ hydrogenation to methanol, the h-PdMo/Mo_2_N catalyst had significantly enhanced catalytic activity and much lower activation energy than the Pd/Mo_2_N catalyst. The methanol synthesis activity of the h-PdMo catalysts was further improved by pressurization, which resulted in continuous CO_2_ hydrogenation to methanol at room temperature. The h-PdMo catalyst had a TOF of 0.15 h^–1^ at 0.9 MPa and 25 °C, which is comparable to or higher than that of the state-of-the-art catalysts under higher-pressure conditions (4–5 MPa). The h-PdMo catalyst also promotes the RWGS reaction above 100 °C. These results indicate that h-PdMo catalysts are more effective for CO_2_ activation and hydrogenation at low temperatures than the Pd catalyst. Controlling the selectivity of products remains a challenge at the next stage. Furthermore, the CO_2_ conversion under the tested conditions (∼1% even at 180 °C) is still far too low compared to the thermodynamic equilibrium value (ca. 10%)^29^ (Table S5). However, the catalytic performance could be much improved by tuning the catalyst structure (e.g., higher surface area, loading on supports other than Mo_2_N, addition of a third metal) and catalytic process. This discovery provides a frontier for catalyst development, not only for low-temperature methanol synthesis and CO_2_ conversion reactions but also for other reactions catalyzed by Pd.

## Supporting Information Available

XRD patterns for the Pd–Mo catalyst and the previously reported PdMo intermetallic, FFT of a single h-PdMo particle, N_2_-TPD profiles for h-PdMo and Mo_2_N, XRD patterns for the h-PdMo catalyst before and after TPD measurements, XRD patterns for the h-PdMo/Mo_2_N and Pd/Mo_2_N catalysts, high-resolution TEM images of h-PdMo/Mo_2_N and Pd/Mo_2_N, FFT of a single h-PdMo nanoparticle and Pd nanoparticle on Mo_2_N, reproducibility test of h-PdMo/Mo_2_N catalyst, H_2_-TPR profile for the h-PdMo/Mo_2_N catalyst, XRD patterns and methanol synthesis activity of h-PdMo/Mo_2_N catalyst synthesized by using Pd(NH_3_)_4_Cl_2_·H_2_O as a Pd source, catalytic activity over Mo_2_N and h-PdMo catalysts under ambient pressure, comparison of catalytic activity over h-PdMo/Mo_2_N and Cu/ZnO/Al_2_O_3_ catalysts, CO_2_ hydrogenation with different flow rates, pressure dependence of catalytic activity over the h-PdMo catalyst, GC-MS spectrum of ^13^CH_3_OH obtained from ^13^CO_2_, catalytic activity and activation energy over the h-PdMo/Mo_2_N and h-PdMo catalysts under pressurized conditions, XRD patterns for the h-PdMo catalyst before and after methanol synthesis, CO-TPD profiles for h-PdMo and Pd, DRIFT spectra over the h-PdMo catalyst, CO hydrogenation to methanol over the h-PdMo/Mo_2_N catalyst under atmospheric pressure, synthesis rate of byproducts and product distribution during CO_2_ hydrogenation, and tables for compositional analysis, structural properties of the studied catalysts, activation energies over various Pd-based catalysts, comparison of TOFs over different catalysts, and CO_2_ conversion over the h-PdMo/Mo_2_N catalyst (PDF)
