# Supplementary material for: Room-Temperature CO2 Hydrogenation to Methanol over Air-Stable hcp-PdMo Intermetallic Catalyst
Source: J Am Chem Soc. 2023 Mar 30;145(17):9410–6. doi: 10.1021/jacs.2c13801 (PMC10161205; doi:10.1021/jacs.2c13801)
Supplement: Supplementary file 1 — ja2c13801_si_001.pdf [file ja2c13801_si_001.pdf]

Supporting Information

Room-Temperature CO_2 Hydrogenation to Methanol over Air-Stable hcp-PdMo Intermetallic Catalyst

Hironobu Sugiyama[1], Masayoshi Miyazaki[1], Masato Sasase[1], Masaaki Kitano[1,2*], Hideo Hosono[1,3*]

[1]MDX Research Center for Element Strategy, International Research Frontiers Initiative Tokyo Institute of Technology, 4259 Nagatsuta, Midori-ku, Yokohama 226-8503, Japan

[2]Advanced Institute for Materials Research (WPI-AIMR), Tohoku University, Sendai 980-8577, Japan

[3]International Center for Materials Nanoarchitectonics (WPI-MANA), National Institute for Materials Science (NIMS), Namiki, Tsukuba, Ibaraki 305-0044, Japan

Correspondence and requests for materials should be addressed to M. Kitano and H. Hosono

* M. Kitano, e-mail: kitano.m.aa@m.titech.ac.jp

* H. Hosono, e-mail: hosono@mces.titech.ac.jp

Experimental section

Preparation of catalysts

h-PdMo, h-PdMo/Mo_2N, and Mo_2N catalysts were prepared *via* ammonolysis of an oxide precursor. Oxide precursors were prepared by the Pechini method using $(NH_4)_6Mo_7O_{24}\cdot 4H_2O$ (81-83% MoO_3 basis, Aldrich) and $Pd(CH_3COO)_2$ (98%, TCI) as Mo and Pd sources, respectively. Mo and Pd sources in predetermined ratios and citric acid at twice the amount of the total metal ions were dissolved in 6% aqueous HNO_3 at room temperature. The solution mixture was placed in a heating mantle at 80 °C, stirred, and evaporated until a transparent gel formed. The temperature was then raised to 200 °C and the gel was converted to an amorphous precursor, which was calcined at 500 °C for 2 h in air to obtain the oxide precursor. Finally, ammonolysis of the oxide precursor was performed at 700 °C in a flow of NH_3 (10 mL min^{-1}) for 12 h. The resultant material with Pd/Mo=1.08 is referred to as h-PdMo, whereas that with Pd/Mo<1.08 is named as h-PdMo/Mo_2N because PdMo intermetallic nanoparticles are formed on Mo_2N as shown in **Figures 2a-e**. Mo_2N was prepared from Mo-oxide without Pd by the same procedure as h-PdMo. To examine the effect of Pd precursor, h-PdMo/Mo_2N (PdMo=0.05) was also synthesized by using $Pd(NH_3)_4Cl_2\cdot H_2O$ (98%, Aldrich) as the Pd source. For Pd/Mo_2N catalyst, $Pd(acac)_2$ (99%, Aldrich) was used as a Pd precursor, which was mixed with Mo_2N in an agate mortar. The mixture was then heated at 300 °C in a flow of H_2 (10 mL min^{-1}) for 2 h with a heating rate of 2 °C min^{-1}. The copper-based methanol synthesis catalyst (Cu/ZnO/Al_2O_3 pellets, Alfa Aesar) was obtained as a commercially available product. Cu/ZnO/Al_2O_3 pellets were hand-milled in an agate mortar and used as a powder. All catalysts were reduced by H_2 at 300 °C for 2 h with a heating rate of 2 °C min^{-1} before the CO_2 hydrogenation reaction.

Methanol synthesis reaction

Methanol synthesis was conducted in a silica-glass fixed-bed reactor with 0.1 g of catalyst in a flow of CO_2-H_2-Ar (CO_2:H_2:Ar = 1:3:1, 50 mL min^{-1}) or CO-H_2-Ar (CO:H_2:Ar = 1:2:1, 40 mL min^{-1}) under atmospheric pressure (0.1 MPa) or CO_2-H_2-Ar (CO_2:H_2:N_2 = 1:3:1, 50 mL min^{-1}) under pressurized conditions (0.3-0.9 MPa). The outlet gas was analyzed using an online gas chromatograph (7890A, Agilent) equipped with thermal conductivity and flame ionization detectors.

Catalyst characterization

The crystal structure was identified using X-ray diffraction (XRD; MiniFlex600, Rigaku or D2 PHASER, Bruker) with Cu Kα radiation (λ = 0.15418 nm). The composition of the PdMo catalyst (Pd/Mo = 1.08) was determined as the average of measurements taken at 50 random points using an electron probe micro analyzer (EPMA; JXA-8530F, Jeol). The amount of Mo and Pd in the catalysts was estimated from inductively coupled plasma atomic emission spectroscopy (ICP-AES; ICPS-8100, Shimadzu) measurements. The morphology and elemental distribution of a single particle of catalyst were evaluated using field-emission scanning electron microscopy (FE-SEM; JSM-7600F, Jeol) with energy-dispersive X-ray spectroscopy (EDX). Temperature-programmed desorption (TPD) of N_2 was conducted by heating (10 °C min^{-1}) a sample in an Ar stream, and the desorbed gas was monitored with a mass spectrometer (BELMass, MicrotracBEL). TPD of CO was performed using the same instrument as TPD of N_2. Prior to measurements, the samples (ca. 30 mg) were reduced under an H_2 flow (10 mL min^{-1}) at 300 °C for 2 h. After cooling to room temperature

with flowing Ar, CO adsorption was conducted in a stream of 10 vol% CO/He at 35 °C. Then, the sample was heated (5 °C min^{-1}) in a stream of Ar and the desorbed gas was monitored with a mass spectrometer (BELMass, MicrotracBEL). Temperature-programmed reaction (TPR) of H_2 was conducted by heating (10 °C min^{-1}) a sample in an H_2 stream, and the desorbed gas was monitored with a mass spectrometer (BELMass, MicrotracBEL). The amount of anions was also estimated with an elemental analyzer (MICRO CORDER JM-10, J-Science). The Brunauer-Emmett-Teller (BET) specific surface areas of the samples were determined from nitrogen adsorption-desorption isotherm measurements at −196 °C using an automatic gas adsorption instrument (BELSORP-mini II, MicrotracBEL). The microstructural characteristics of the samples were determined using transmission electron microscopy (TEM; JEM-ARM200F, Jeol). EDX mapping of the same area was also performed. Diffuse reflectance infrared Fourier transform (DRIFT) spectroscopy was performed using a spectrometer (FT/IR-6X, Jasco) with a mercury-cadmium-tellurium (MCT) detector at a resolution of 4 cm^{-1}. Prior to CO_2 hydrogenation, the samples were reduced by H_2 at 300 °C for 2 h. After the sample was cooled to room temperature, a mixed gas (CO_2:H_2 = 1:3, 20 mL min^{-1}) was supplied to the chamber, and measurements were conducted at room temperature.

Supplementary Note 1: Stabilization of hcp-PdMo intermetallic phase

According to the equilibrium diagram of the Pd-Mo system, an intermetallic compound with the hexagonal close-packed (hcp) structure exists in the range of 50-60 at% Pd.[1] The XRD pattern for this phase (ICSD No. 105061) is very close to that of the present sample (Pd = 52 at%) (**Figure S1**). In the XRD pattern of this sample, peaks except for the 002 plane are shifted to lower angle, which indicates distortion and expansion of the lattice due to the insertion of anions into the interstitial sites, as confirmed by compositional analysis (**Table S1**). HAADF-STEM observation revealed that Pd and Mo were distributed in alternating layers perpendicular to the C-axis direction (**Figure 1b and S2**). Such an ordered structure is considered to be responsible for the anisotropic lattice expansion. The PdMo phase containing anions is referred to as h-PdMo and is distinguished from the PdMo intermetallic without anions. The PdMo intermetallic generally decomposes into Mo (body-centered cubic (bcc) structure) and Pd (face-centered cubic (fcc) structure) below 1450 °C. Therefore, it is impossible to synthesize or stabilize this phase at lower temperatures. In our experiments, we speculate that the insertion of anions into the interstitial sites during the ammonolysis process enabled the synthesis and stabilization of this phase at lower temperatures (≤700 °C). N_2 desorption temperature from h-PdMo was lower than that from Mo_2N (**Figure S3**), which suggests that nitrogen is surrounded by not only Mo but also Pd. When the anions were desorbed by TPD, the h-PdMo sample decomposed into Mo and Pd, as shown in **Figure S4**.

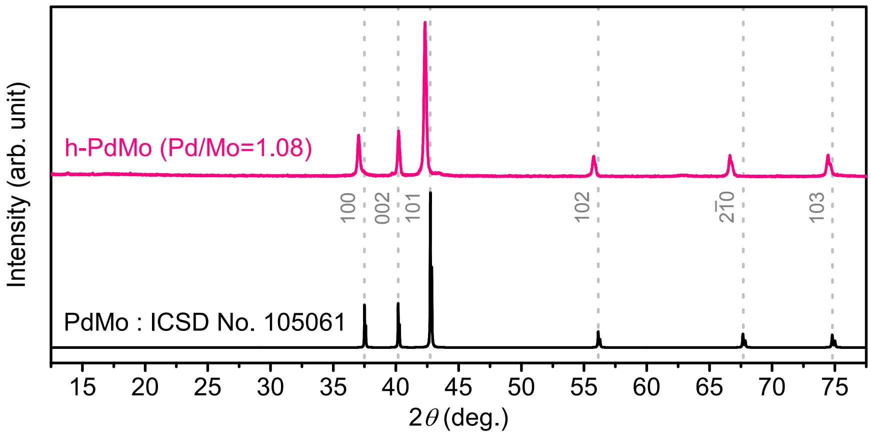

Figure S1 Comparison of XRD patterns for the Pd-Mo catalyst and the previously reported PdMo intermetallic.

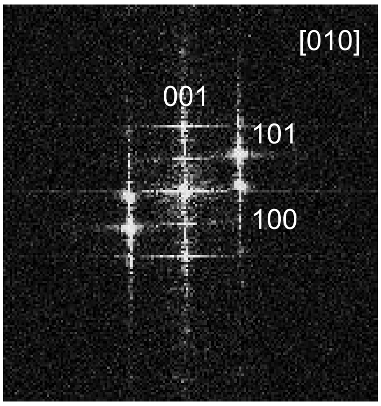

Figure S2 Fast Fourier transform (FFT) of a single h-PdMo particle. The FFT of the TEM image revealed a set of diffraction spots corresponding to a hcp-like crystal oriented along the [010] direction.

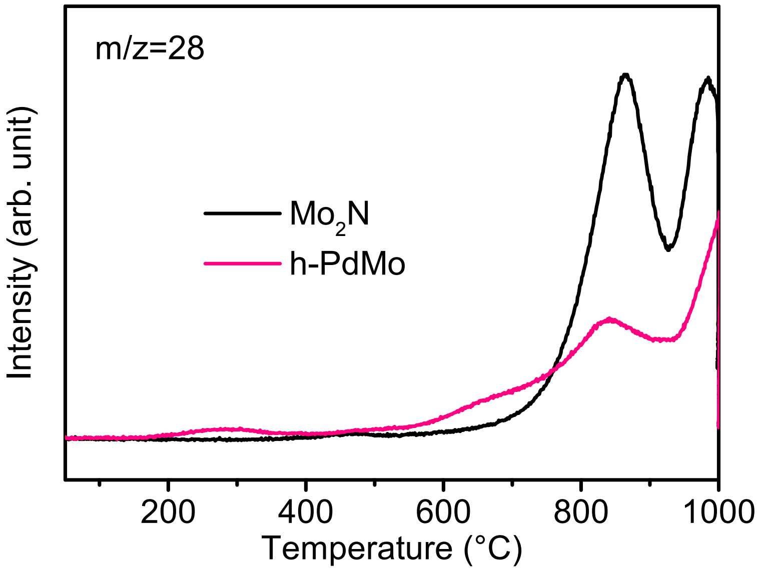

Figure S3 N_2-TPD profiles for h-PdMo and Mo_2N. The TPD experiment was performed with a 10 mL min^{-1} flow of Ar.

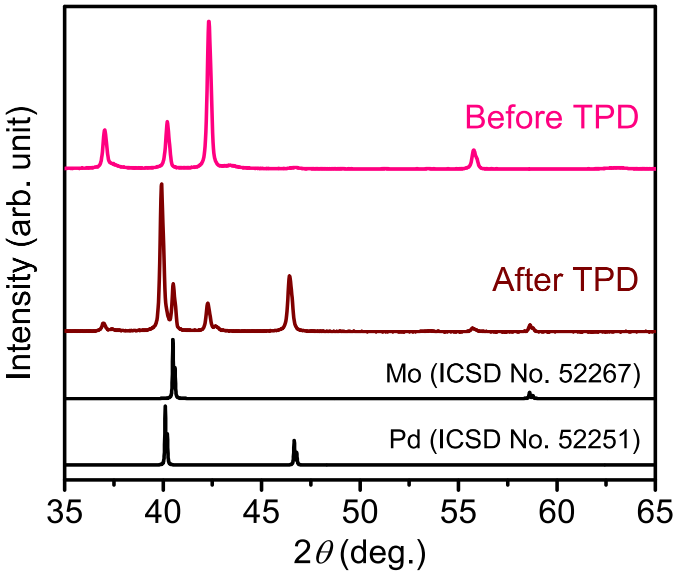

Figure S4 XRD patterns for the h-PdMo catalyst before and after TPD measurements.

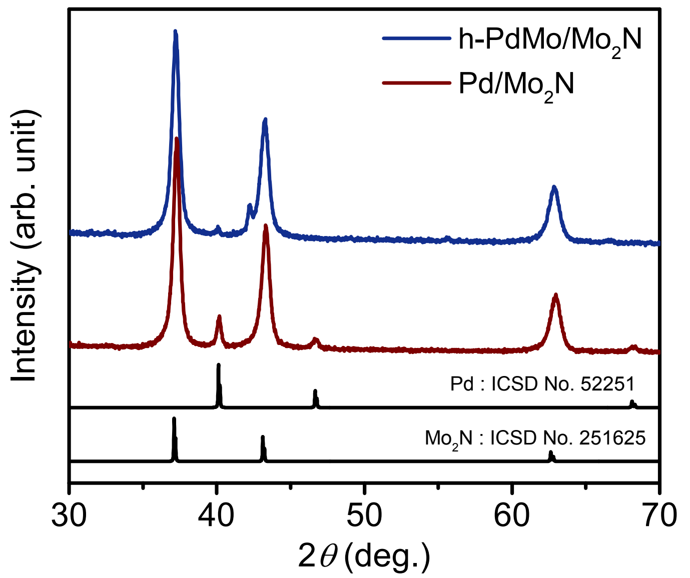

Figure S5 XRD patterns for the h-PdMo/Mo_2N and Pd/Mo_2N catalysts.

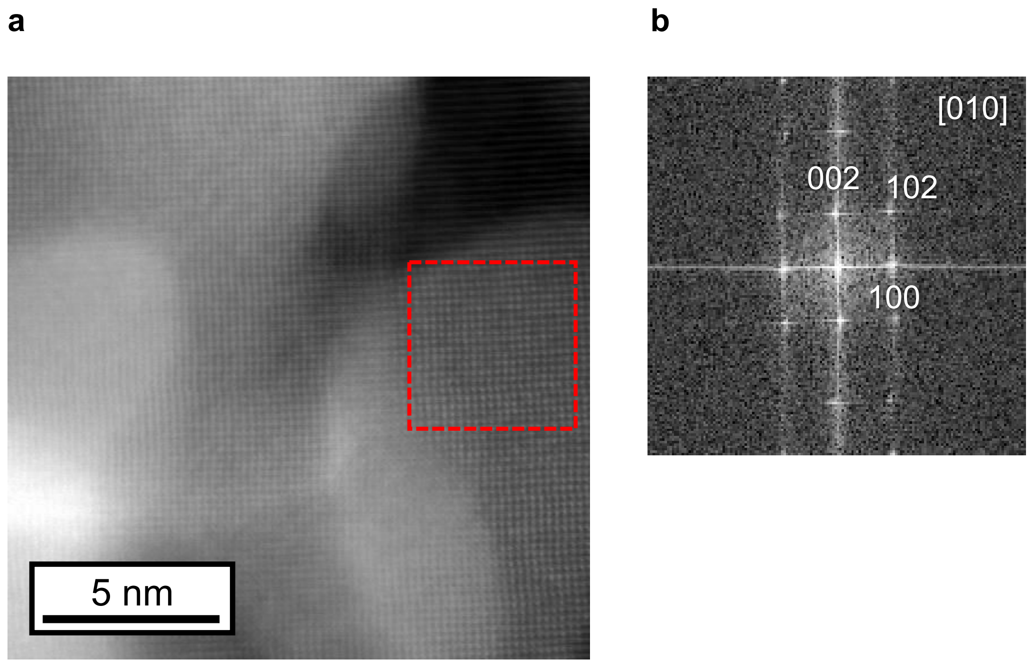

Figure S6 (a) High-resolution TEM image of h-PdMo/Mo_2N. (b) FFT of a single h-PdMo nanoparticle embedded on Mo_2N in (a). The FFT of the TEM image revealed a set of diffraction spots corresponding to a hcp-like crystal oriented along the [010] direction.

a b

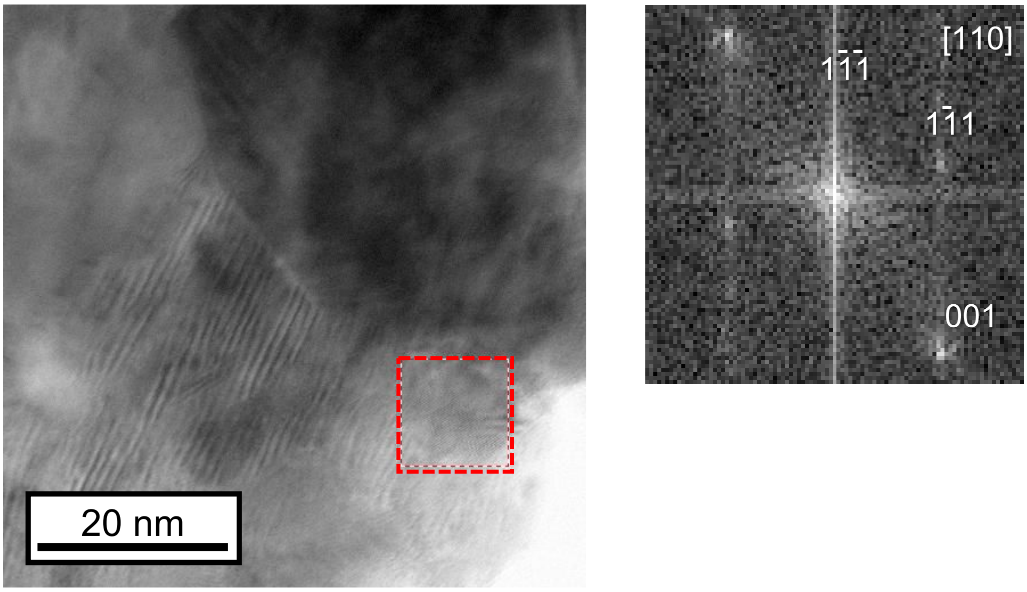

Figure S7 (a) High-resolution TEM image of Pd/Mo_2N. (b) FFT of a single Pd nanoparticle embedded on Mo_2N in (a). The FFT of the TEM image revealed a set of diffraction spots corresponding to a fcc crystal oriented along the [110] direction.

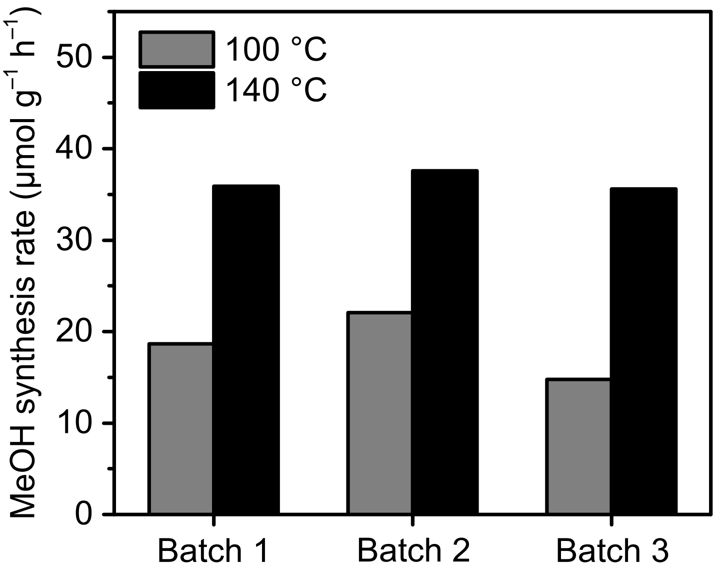

Figure S8 Reproducibility test of h-PdMo/Mo_2N catalyst. Standard deviation from three independent catalysts is below ±3 μmol g^{-1} h^{-1}. (Reaction conditions: 0.1 g catalyst, CO_2:H_2:Ar = 10:30:10 mL min^{-1}, 100 or 140 °C, 0.1 MPa).

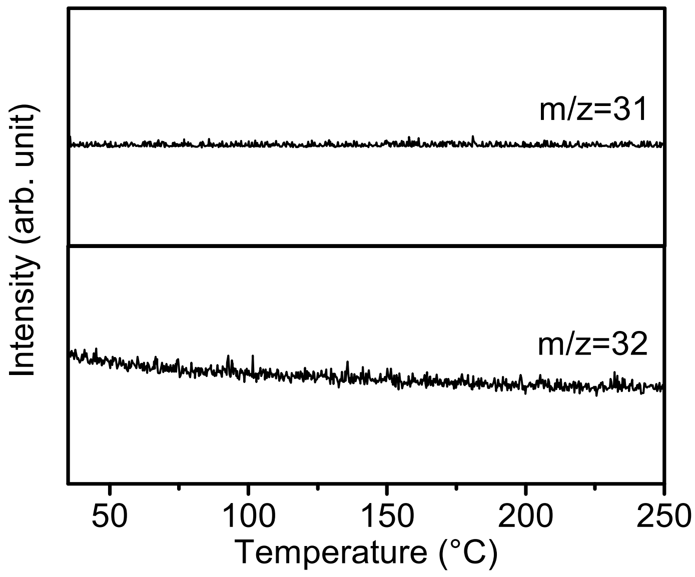

Figure S9 H_2-TPR profile for the h-PdMo/Mo_2N catalyst.

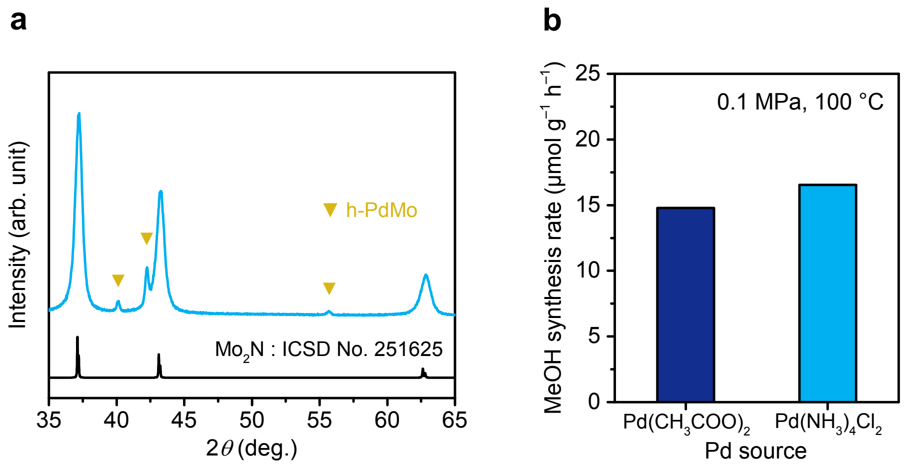

Figure S10 (a) XRD patterns and (b) methanol synthesis activity of h-PdMo/Mo_2N (Pd/Mo=0.05) catalyst synthesized by using $Pd(NH_3)_4Cl_2{\cdot}H_2O$ as the Pd source. (Reaction conditions: 0.1 g catalyst, CO_2:H_2:Ar = 10:30:10 mL min^{-1}, 100 °C, 0.1 MPa).

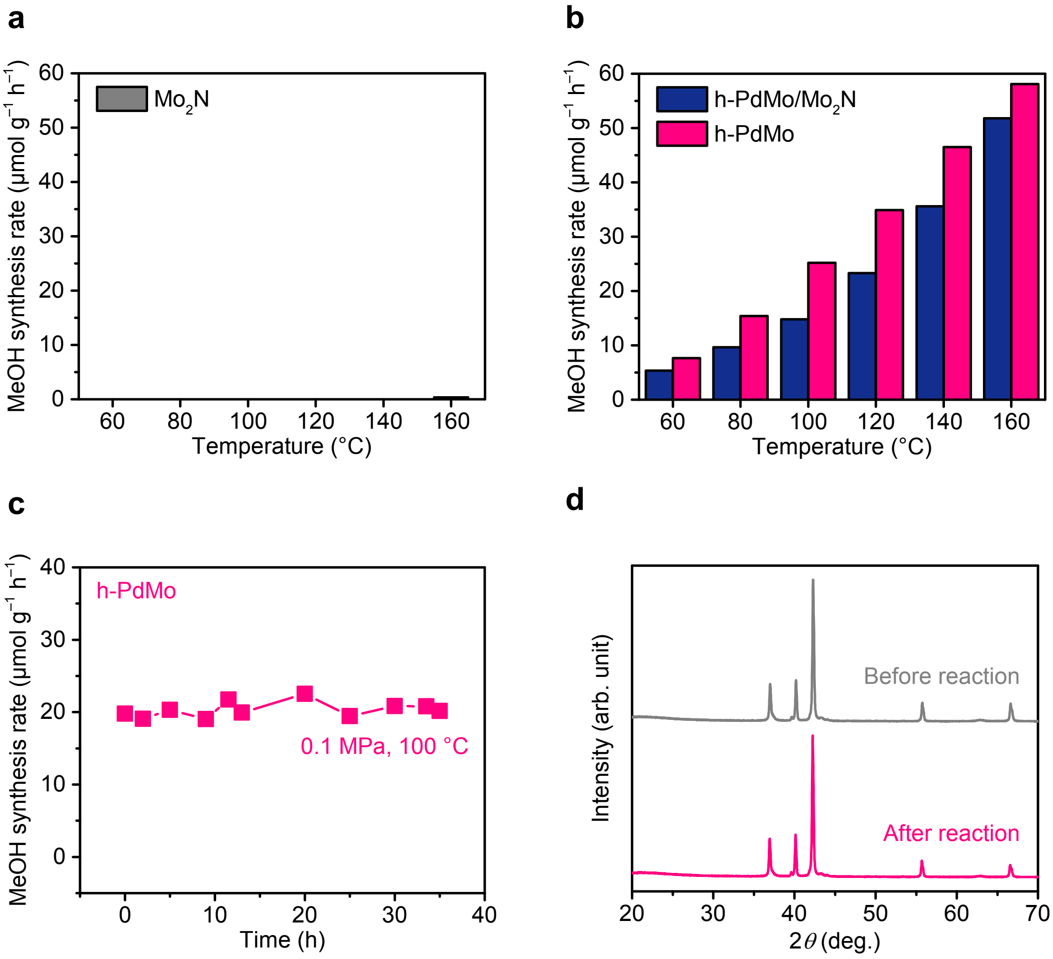

Figure S11 Catalytic activity of CO_2 hydrogenation to methanol over (a) Mo_2N and (b-d) h-PdMo catalysts under ambient pressure. (a, b) Methanol synthesis rate as a function of reaction temperature. (c) Time course at 100 °C. (d) XRD patterns before and after methanol synthesis at 100 °C for 35 h. The h-PdMo catalyst with a Pd/Mo ratio of 1.08 was almost single phase hcp-PdMo (Fig. 1a). (Reaction conditions: 0.1 g catalyst, CO_2:H_2:Ar = 10:30:10 mL min^{-1}, 60-160 °C, 0.1 MPa).

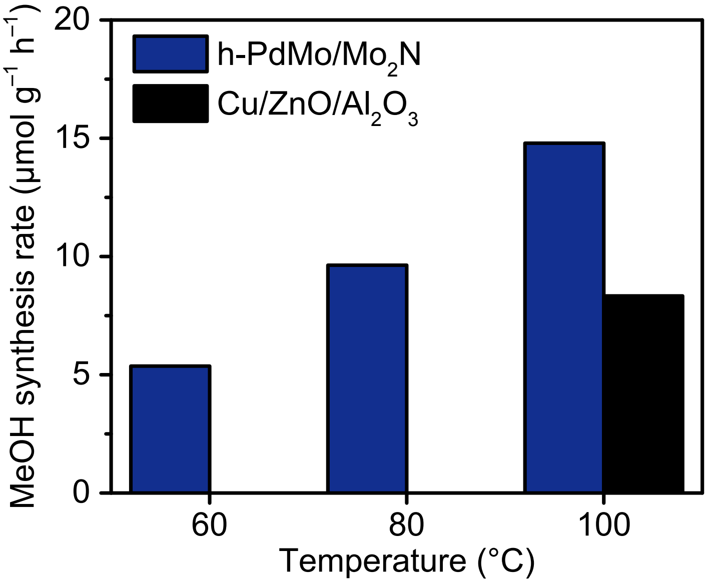

Figure S12 Comparison of catalytic activity for CO_2 hydrogenation to methanol over h-PdMo/Mo_2N and commercial Cu/ZnO/Al_2O_3 catalysts. (Reaction conditions: 0.1 g catalyst, CO_2:H_2:Ar = 10:30:10 mL min^{-1}, 60-100 °C, 0.1 MPa).

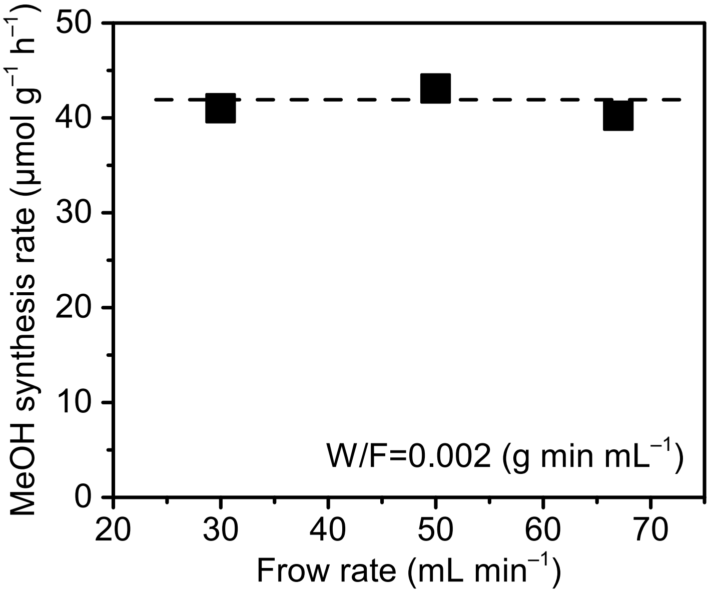

Figure S13 CO_2 hydrogenation to methanol over h-PdMo/Mo_2N catalyst with fixed W/F and varying flow rates. (Reaction conditions: 0.06-0.13 g catalyst, flow rate = 30-67 mL min^{-1} (CO_2:H_2:Ar = 1:3:1), 140 °C, 0.1 MPa).

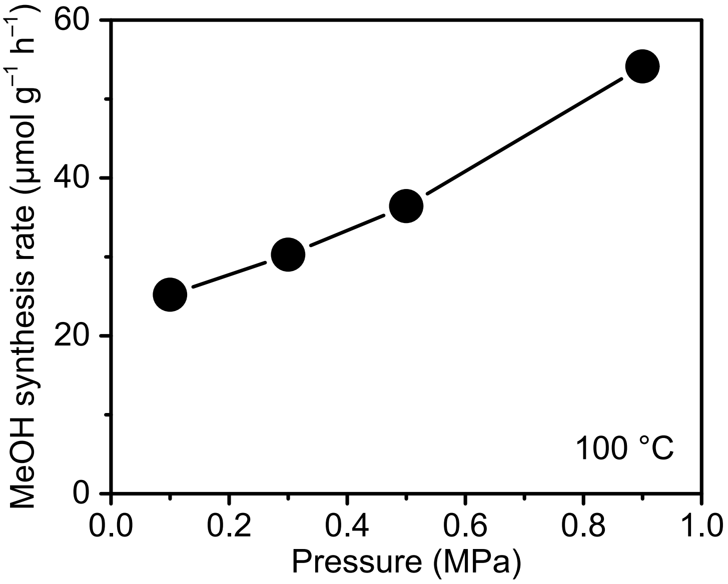

Figure S14 Pressure dependence of catalytic activity for CO_2 hydrogenation to methanol over the h-PdMo catalyst.

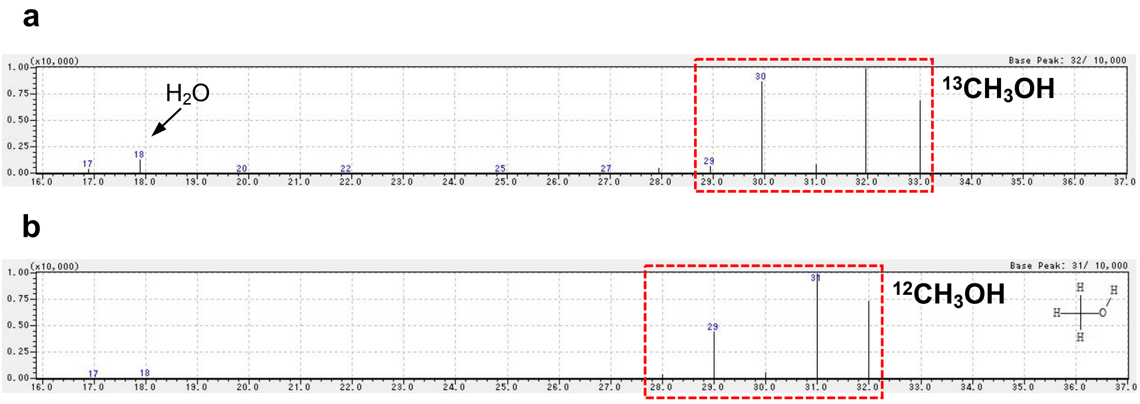

Figure S15 GC-MS spectra of (a) $^{13}CH_3OH$ obtained from $^{13}CO_2$ hydrogenation, and (b) $^{12}CH_3OH$ as a reference. Hydrogenation of $^{13}CO_2$ (purity 99%) to $^{13}CH_3OH$ was conducted using a 25 mL stainless steel autoclave equipped with a manometer. The autoclave was flushed five times with H_2, and then the mixture of $^{13}CO_2$ and H_2 gases (total pressure: 1 MPa, $^{13}CO_2$:H_2 = 1:3) was introduced into the reaction system. The reaction was performed with h-PdMo catalyst (ca. 400 mg) at 35 °C for 24 h. After the reaction, the pressurized gases were introduced into the water, and the $^{13}CH_3OH$ trapped in the water was analyzed by GC-MS (GCMS-QP2020 NX, Shimadzu).

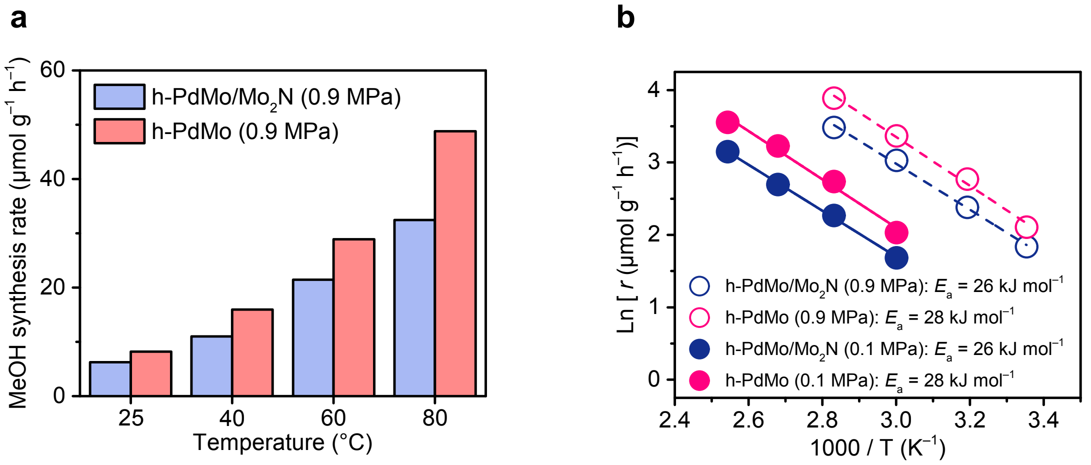

Figure S16 Comparison of (a) catalytic activity and (b) apparent activation energy for CO_2 hydrogenation to methanol over the h-PdMo/Mo_2N and h-PdMo catalysts under pressurized conditions (0.9 MPa). (Reaction conditions: 0.1 g catalyst, CO_2:H_2:N_2 = 10:30:10 mL min^{-1}, 25-80 °C, 0.9 MPa). The apparent activation energy under ambient pressure is also shown in (b) for comparison.

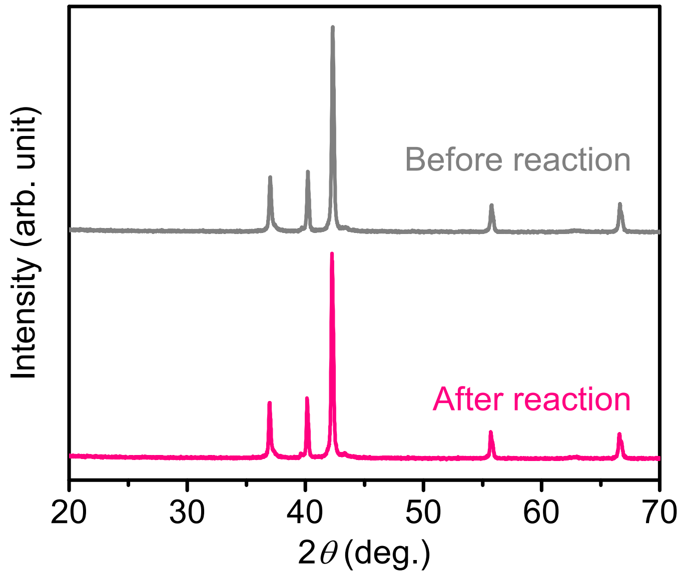

Figure S17 XRD patterns for the h-PdMo catalyst before and after methanol synthesis at 0.9 MPa and 25 °C for 50 h.

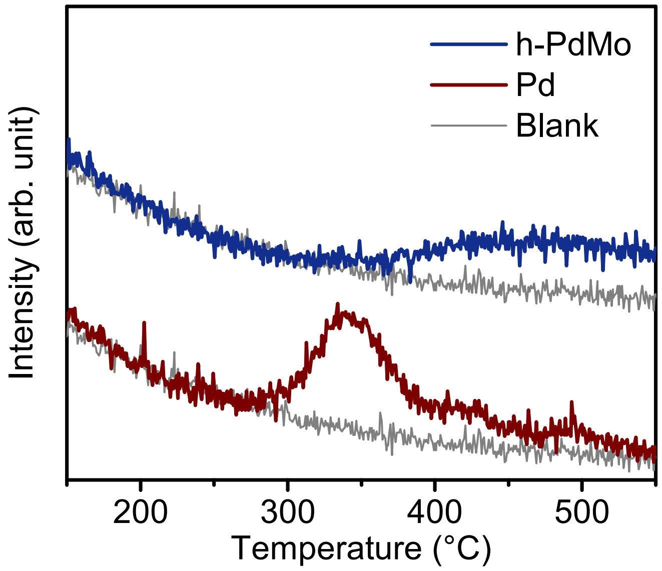

Figure S18 CO-TPD profiles for h-PdMo and Pd. The TPD experiment was performed with a 10 mL min^{-1} flow of Ar after CO adsorption at 35 °C.

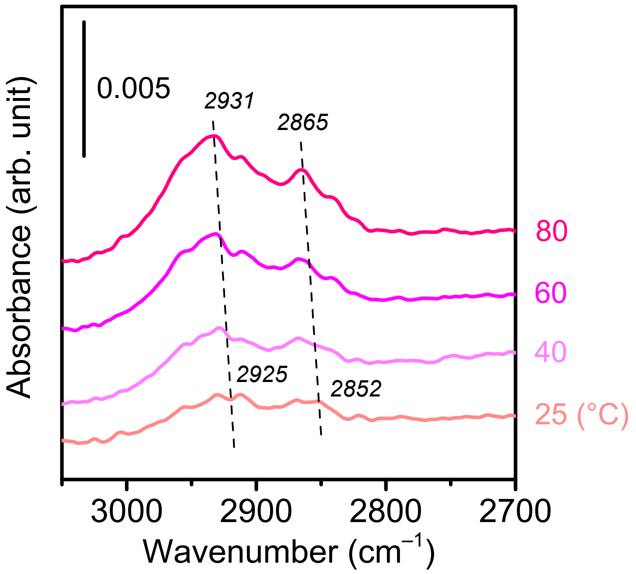

Figure S19 DRIFT spectra for CO_2 hydrogenation over the h-PdMo catalyst at various temperatures (25-80 °C).

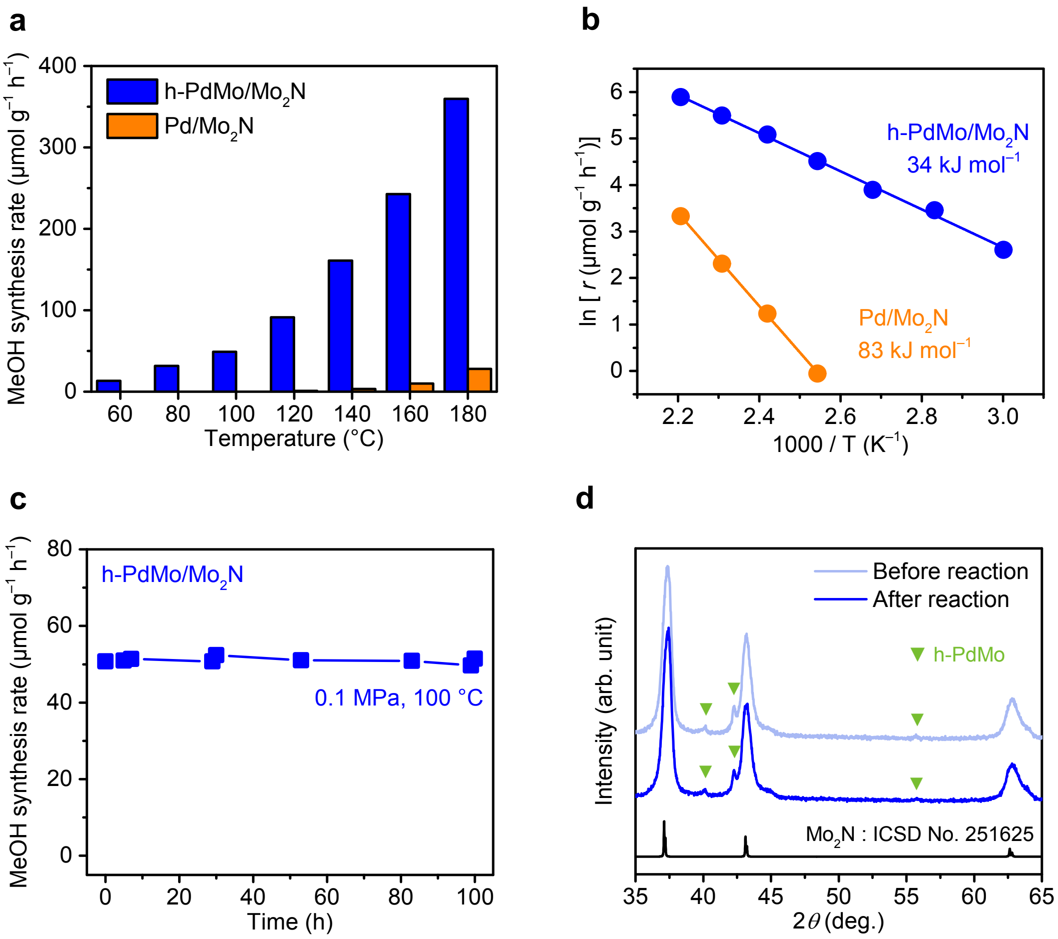

Figure S20 CO hydrogenation to methanol over the h-PdMo/Mo_2N catalyst under atmospheric pressure. (a) Methanol synthesis rate as a function of reaction temperature. (b) Arrhenius plots for methanol synthesis. (c) Time course at 100 °C. (d) XRD patterns before and after methanol synthesis at 100 °C for 100 h.

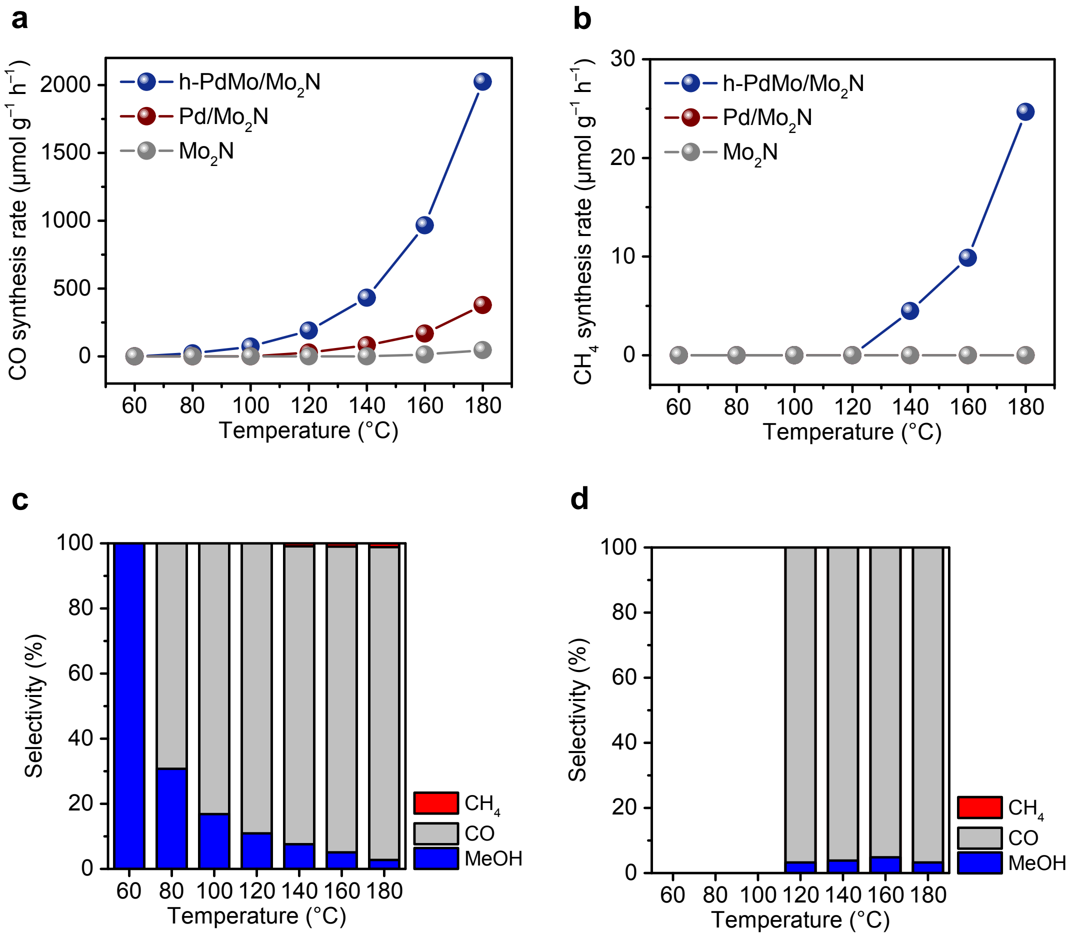

Figure S21 Synthesis rate of byproducts and product distribution during CO_2 hydrogenation over h-PdMo/Mo_2N and Pd/Mo_2N catalysts. (a) CO and (b) CH_4 synthesis rate as a function of reaction temperature. Product distribution during CO_2 hydrogenation over (c) h-PdMo/Mo_2N and (d) Pd/Mo_2N catalysts. Selectivity of products was determined by eq 1.

$$\text{Selectivity of x species (\%)} = 100 \times F_{x,out} / (F_{methanol,out} + F_{CO,out} + F_{CH4,out}) \qquad (1)$$

where $F_{x,out}$ is the outlet flow rate of products (ml min^{-1}). (Reaction conditions: 0.1 g catalyst, CO_2:H_2:Ar = 10:30:10 mL min^{-1}, 60-180 °C, 0.1 MPa).

Table S1 Compositional analysis results for the single phase sample (Pd/Mo = 1.08).

				(wt %)
	Pd	Mo	N	O
EPMA	58.1	39.2	1.1	1.6
ICP-AES	51.3	44.5	-	-
TPD	-	-	1.9	-
CHN	-	-	2.0	1.8
				(mol%)
EPMA	48.2	36.1	6.9	8.9

Table. S2 Structural properties of the studied catalysts.

	Amount of Pd (wt%)*	Surface area ($m^2\ g^{-1}$)
h-PdMo	51.3	2.6
h-PdMo/Mo_2N	4.4	12.9
Pd/Mo_2N	4.6	25.1

*Amount of Pd in each catalyst was determined by ICP-AES measurement.

Table. S3 Apparent activation energies for CO_2 hydrogenation to methanol over various Pd-based catalysts.

	Activation energy (kJ mol^{-1})	Reference
h-PdMo/Mo_2N	27	This work
Pd/Mo_2N	78	This work
Pd/SiO_2	65	[2]
Pd-Zn/SiO_2	58	[3]
Pd-Ga/SiO_2	48	[3]
Ga_2O_3-Pd/SiO_2	37-39	[4]
$Pd_{0.1}Zn_1$/CNT	57	[5]
$Pd_{0.1}Zn_1$/Al_2O_3	65	[5]
Pd/In_2O_3	84	[6]
Pd (*PdMgAl* HTlc)*	72	[7]
PdZn (*PdZnAl* HTlc)†	68	[7]
Pd_2Ga (*PdMgGa* HTlc)‡	59	[7]

*Pd-loaded catalyst derived from *PdMgAl* hydrotalcite. †PdZn-loaded catalyst derived from *PdZnAl* hydrotalcite. ‡Pd_2Ga-loaded catalyst derived from *PdMgGa* hydrotalcite.

Table S4 TOFs over the h-PdMo catalyst, other reported room-temperature methanol synthesis catalysts, and conventional Cu or Pd-based catalysts.

Catalyst	*T* (ºC)	*P* (MPa)	TOF (h^{-1})	Reference
h-PdMo	25	0.9	0.15*	This work
Ir complex	30	4	0.02†	[8]
FL-MoS_2	25	5	0.09-0.52‡	[9]
$Cu/ZnO/Al_2O_3$	100	7	0.05†	[10]
Pd/CNT	250	2	0.33†	[11]
Pd/SiO_2	250	2	0.06†	[11]
Pd/AC	250	2	0.11†	[11]

*TOF calculated from the rate of methanol synthesis divided by the total metal sites. The total metal sites was estimated from the BET surface area, assuming that all surfaces are metal atoms (Pd:Mo = 6:4). †TOF calculated from the rate of methanol synthesis divided by total metal (Ir, Cu, or Pd) sites. ‡TOF calculated based on a method in the literature.[9] The highest and lowest TOFs were calculated on the basis of the amount of exposed sulfur vacancies or Mo atoms. The average value of the highest and lowest TOFs was then calculated as the TOF of the FL-MoS_2 catalyst.

Table. S5 CO_2 conversion over the h-PdMo/Mo_2N catalyst under different reaction conditions.

P (MPa)	T (°C)	Conversion (%)*
0.1	60	0.002
0.1	80	0.014
0.1	100	0.036
0.1	120	0.096 (0.104)
0.1	140	0.211 (0.207)
0.1	160	0.460 (0.467)
0.1	180	0.943 (0.939)
0.9	25	0.004
0.9	40	0.006
0.9	60	0.012
0.9	80	0.020

*Since it is difficult to quantify tiny changes in CO_2 flow rate especially in reactions below 100°C, CO_2 conversion was determined by eq 2.

$$CO_2 \text{ conversion (\%)} = 100 \times (F_{\text{methanol,out}} + F_{\text{CO,out}}) / F_{\text{CO2,in}} \qquad (2)$$

where $F_{\text{CO2,in}}$ is the inlet flow rate of CO_2 (ml min^{-1}) and $F_{\text{x,out}}$ is the outlet flow rate of products (ml min^{-1}). The parentheses indicate the CO_2 conversion determined by eq 3.

$$CO_2 \text{ conversion (\%)} = 100 \times (F_{\text{CO2,in}} - F_{\text{CO2,out}}) / F_{\text{CO2,in}} \qquad (3)$$

The values of the CO_2 conversion obtained from both equations are comparable, and the mass balances for carbon generally fulfilled.

References

(1) Anderson, E. The Equilibrium Diagram of the System Molybdenum-Palladium. *Journal of the Less Common Metals* **1964**, *6* (1), 81–84. https://doi.org/10.1016/0022-5088(64)90009-8.

(2) Manrique, R.; Rodríguez-Pereira, J.; Rincón-Ortiz, S. A.; Bravo-Suárez, J. J.; Baldovino-Medrano, V. G.; Jiménez, R.; Karelovic, A. The Nature of the Active Sites of Pd–Ga Catalysts in the Hydrogenation of CO_2 to Methanol. *Catal. Sci. Technol.* **2020**, *10* (19), 6644–6658. https://doi.org/10.1039/D0CY00956C.

(3) Manrique, R.; Jiménez, R.; Rodríguez-Pereira, J.; Baldovino-Medrano, V. G.; Karelovic, A. Insights into the Role of Zn and Ga in the Hydrogenation of CO_2 to Methanol over Pd. *International Journal of Hydrogen Energy* **2019**, *44* (31), 16526–16536. https://doi.org/10.1016/j.ijhydene.2019.04.206.

(4) Collins, S. E.; Baltanás, M. A.; Delgado, J. J.; Borgna, A.; Bonivardi, A. L. CO_2 Hydrogenation to Methanol on Ga_2O_3-Pd/SiO_2 Catalysts: Dual Oxide-Metal Sites or (Bi)Metallic Surface Sites? *Catalysis Today* **2021**, *381*, 154–162. https://doi.org/10.1016/j.cattod.2020.07.048.

(5) Liang, X.-L.; Dong, X.; Lin, G.-D.; Zhang, H.-B. Carbon Nanotube-Supported Pd–ZnO Catalyst for Hydrogenation of CO_2 to Methanol. *Applied Catalysis B: Environmental* **2009**, *88* (3), 315–322. https://doi.org/10.1016/j.apcatb.2008.11.018.

(6) Frei, M. S.; Mondelli, C.; García-Muelas, R.; Kley, K. S.; Puértolas, B.; López, N.; Safonova, O. V.; Stewart, J. A.; Curulla Ferré, D.; Pérez-Ramírez, J. Atomic-Scale Engineering of Indium Oxide Promotion by Palladium for Methanol Production via CO_2 Hydrogenation. *Nat Commun* **2019**, *10* (1), 3377. https://doi.org/10.1038/s41467-019-11349-9.

(7) Ota, A.; Kunkes, E. L.; Kasatkin, I.; Groppo, E.; Ferri, D.; Poceiro, B.; Navarro Yerga, R. M.; Behrens, M. Comparative Study of Hydrotalcite-Derived Supported Pd_2Ga and PdZn Intermetallic Nanoparticles as Methanol Synthesis and Methanol Steam Reforming Catalysts. *Journal of Catalysis* **2012**, *293*, 27–38. https://doi.org/10.1016/j.jcat.2012.05.020.

(8) Kanega, R.; Onishi, N.; Tanaka, S.; Kishimoto, H.; Himeda, Y. Catalytic Hydrogenation of CO_2 to Methanol Using Multinuclear Iridium Complexes in a Gas–Solid Phase Reaction. *J. Am. Chem. Soc.* **2021**, *143* (3), 1570–1576. https://doi.org/10.1021/jacs.0c11927.

(9) Hu, J.; Yu, L.; Deng, J.; Wang, Y.; Cheng, K.; Ma, C.; Zhang, Q.; Wen, W.; Yu, S.; Pan, Y.; Yang, J.; Ma, H.; Qi, F.; Wang, Y.; Zheng, Y.; Chen, M.; Huang, R.;

Zhang, S.; Zhao, Z.; Mao, J.; Meng, X.; Ji, Q.; Hou, G.; Han, X.; Bao, X.; Wang, Y.; Deng, D. Sulfur Vacancy-Rich MoS_2 as a Catalyst for the Hydrogenation of CO_2 to Methanol. *Nat Catal* **2021**, *4* (3), 242–250. https://doi.org/10.1038/s41929-021-00584-3.

(10) Reller, C.; Pöge, M.; Lißner, A.; Mertens, F. O. R. L. Methanol from CO_2 by Organo-Cocatalysis: CO_2 Capture and Hydrogenation in One Process Step. *Environ. Sci. Technol.* **2014**, *48* (24), 14799–14804. https://doi.org/10.1021/es503914d.

(11) Wang, J.; Lu, S.; Li, J.; Li, C. A Remarkable Difference in CO_2 Hydrogenation to Methanol on Pd Nanoparticles Supported inside and Outside of Carbon Nanotubes. *Chem. Commun.* **2015**, *51* (99), 17615–17618. https://doi.org/10.1039/C5CC07079A.
